# Just-in-time faculty development: a mobile application helps clinical teachers verify and describe clinical reasoning difficulties

**DOI:** 10.1186/s12909-019-1558-2

**Published:** 2019-04-30

**Authors:** Elisabeth Boileau, Marie-Claude Audétat, Christina St-Onge

**Affiliations:** 1 Department of Family and Emergency Medicine, Université de Sherbrooke, Sherbrooke, Canada; 2 Faculty of Medicine, Université de Genève, Geneva, Switzerland; 3 Faculty of Medicine, Université de Montréal, Montreal, Canada; 4 Department of Medicine, Université de Sherbrooke, Sherbrooke, Canada; 5 Paul Grand’Maison de la SMUS, Université de Sherbrooke, 3001, 12e avenue N, Sherbrooke, QC J1H 5N4 Canada

**Keywords:** Clinical reasoning, Clinical supervision, Mobile technology, Underperformance

## Abstract

**Background:**

Although clinical teachers can often identify struggling learners readily and reliably, they can be reluctant to act upon their impressions, resulting in failure to fail. In the absence of a clear process for identifying and remediating struggling learners, clinical teachers can be put off by the prospect of navigating the politically and personally charged waters of remediation and potential failing of students.

**Methods:**

To address this gap, we developed a problem-solving algorithm to support clinical teachers from the identification through the remediation of learners with clinical reasoning difficulties, which have significant implications for patient care. Based on this algorithm, a mobile application (Pdx) was developed and assessed in two emergency departments at a Canadian university, from 2015 to 2016, using interpretive description as our research design. Semi-structured interviews were conducted before and after a three-month trial with the application. Interviews were analysed both deductively, using pre-determined categories, and inductively, using emerging categories.

**Results:**

Twelve clinical teachers were interviewed. Their experience with the application revealed their need to first validate their impressions of difficulties in learners and to find the right words to describe them before difficulties could be addressed. The application was unanimously considered helpful regarding both these aspects, while the mobile format appeared instrumental in allowing clinical teachers to quickly access targeted information during clinical supervision.

**Conclusions:**

The value placed on verifying impressions and finding the right words to pinpoint difficulties should be further explored in endeavours that aim to address the failure to fail phenomenon. Moreover, just-in-time mobile solutions, which mirror habitual clinical practices, may be used profitably for knowledge transfer in medical education, as an alternative form of faculty development.

**Electronic supplementary material:**

The online version of this article (10.1186/s12909-019-1558-2) contains supplementary material, which is available to authorized users.

## Background

Clinical teachers are exposed to learners’ actual performances when they supervise them in a real clinical setting [1]. In this context, clinical teachers are generally able to identify struggling learners readily and spontaneously, and they have been shown to do so reliably [2]. When learners’ difficulties are addressed early by clinical teachers during clerkships, more time remains for adequate remediation [3]. In addition, in-training remediation that is integrated to regular clinical activities and supervised by clinical teachers is often regarded as more effective and time-efficient than extracurricular activities [1]. Early identification and remediation of learners’ difficulties by clinical teachers have therefore been described as a best practice when supervising learners in clinical settings [4].

Conversely, failure to address learners’ difficulties causes delays in remediation which sometimes allows critical incidents to occur with tangible consequences for patients before a red flag is raised [3, 5]. This situation is often referred to as failure to fail and occurs as clinical teachers are often reluctant to address learners’ difficulties in the absence of clear or familiar steps to follow once a learner in difficulty is identified [6]. Because clinical teachers can be put off by the prospect of having to navigate the politically and personally charged waters of remediation and potential failing of students, they often express their wish to be guided through these steps, particularly with respect to the first steps before formally identifying a problem [7].

Clinical teachers have expressed a need for guidance when addressing clinical reasoning difficulties more specifically [8]. These difficulties are among the most frequent causes of clinical underperformance [5] and while they lead to errors in diagnosis and treatment with potentially serious consequences, they also have a good prognosis for remediation [9]. However, when asked to supervise learners who presented such difficulties, clinical teachers surveyed by Audetat et al. [8] explicitly expressed a wish to have “a tool that would tell them what to do”.

Yepes et al. [10] identified “unsatisfactory evaluator development and evaluation tools as a barrier to failing underperforming trainees”, calling on “health professions educators to develop effective solutions” to address such barriers (p.1092–1093). To date, a few solutions have been explored to facilitate clinical teachers’ task of assessing trainee performance accurately during clinical supervision. In the existing literature, suggested solutions aimed at the clinical teacher level have mainly revolved around targeting areas for faculty development [6, 11]. While one study has demonstrated increased knowledge on how to supervise struggling learners after a faculty development workshop [12], much less is known about how such knowledge later translates into practice [13]. Another suggested solution has been to give clinical teachers feedback on their assessment of trainees [14], and one study did in fact find that the quality of documented assessment could be improved by giving clinical teachers repeated feedback [15]. None of these solutions has addressed all steps of the clinical teacher’s task from identification to remediation strategies nor explored actual use during supervision.

This article describes one endeavour to fill this gap by developing a mobile application designed to guide clinical teachers in the clinical supervision of learners with clinical reasoning difficulties. The assessment of clinical reasoning difficulties during clinical supervision is a multiple assessments and lower stake context for the evaluation of learners. Use of an educational tool by clinical teachers in such a context is generally acknowledged to be based on their subjective perceptions of the tool and its coherence with their usual practices, rather than on the psychometric qualities of the tool [16, 17]. For this reason, clinical teachers’ impressions of the application in this study were explored qualitatively. In addition, in order to foster greater coherence with clinical teachers’ usual practices, we designed our educational tool so as to draw an explicit parallel between clinical and educational diagnostic approaches. Indeed, both diagnostic approaches are aimed at problem-solving [18], using similar steps [19] and strategies [20], based on specialized knowledge organized in scripts [21–24].

Because our tool was developed to assess the clinical reasoning competence of medical trainees, our explorative approach was driven by Van der Vleuten’s conceptual framework for gauging tools used in the assessment of professional competence in medical education [16]. In this conceptual framework, the perceived utility of a tool is determined by its educational impact and acceptability to intended users, in addition to the tool’s validity and reliability. Considering that users’ subjective perception of a tool is the main predictor of actual use in such a low stake context with multiple assessors [16, 17], this study focused on the acceptability of the tool. According to Van der Vleuten, acceptability is influenced by the opinions, sentiments and traditions of teachers regarding a tool and by the perceived feasibility of using an educational tool within time constraints [16, 25].

## Methods

**Fig. 1:**
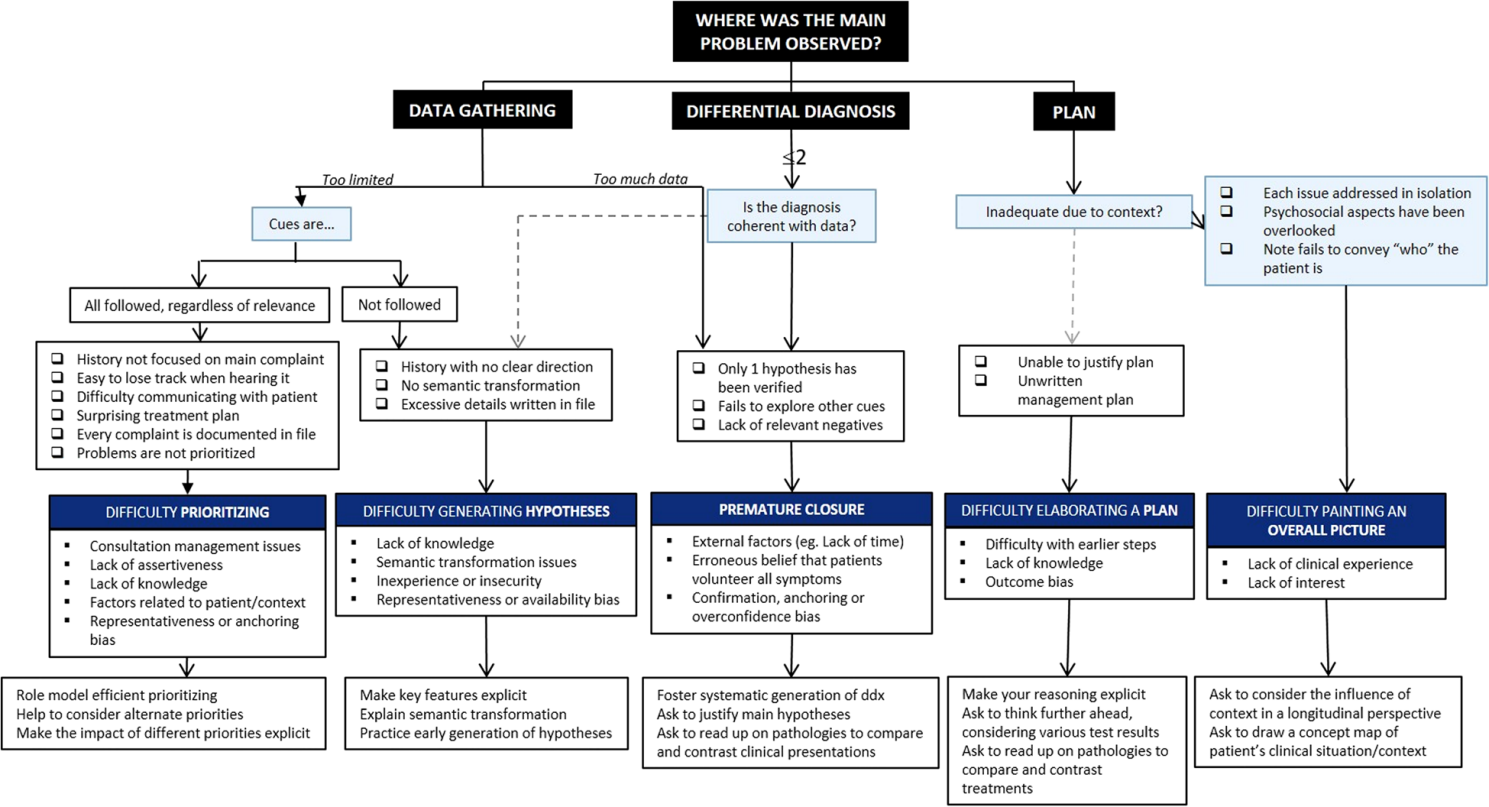
Decision tree based on Audetat et al.’s taxonomy of clinical reasoning difficulties

Audetat et al. [8] have developed a reference guide and taxonomy of the five most frequent prototypes of clinical reasoning difficulties. Based on this guide, we first elaborated a decision tree. While the reference guide addresses the five prototypes separately, listing their respective signs, differential diagnoses and remediation strategies, our decision tree groups the five prototypes in one figure (Fig. 1). To increase ease of use during supervision, the decision tree was designed to begin with familiar signs of clinical reasoning difficulties as entry points, in the way that they would first be observed by clinical teachers in the field. Depending on their answers, clinical teachers are led by arrows to the relevant pedagogical diagnoses and remediation strategies.

**Fig. 2:**
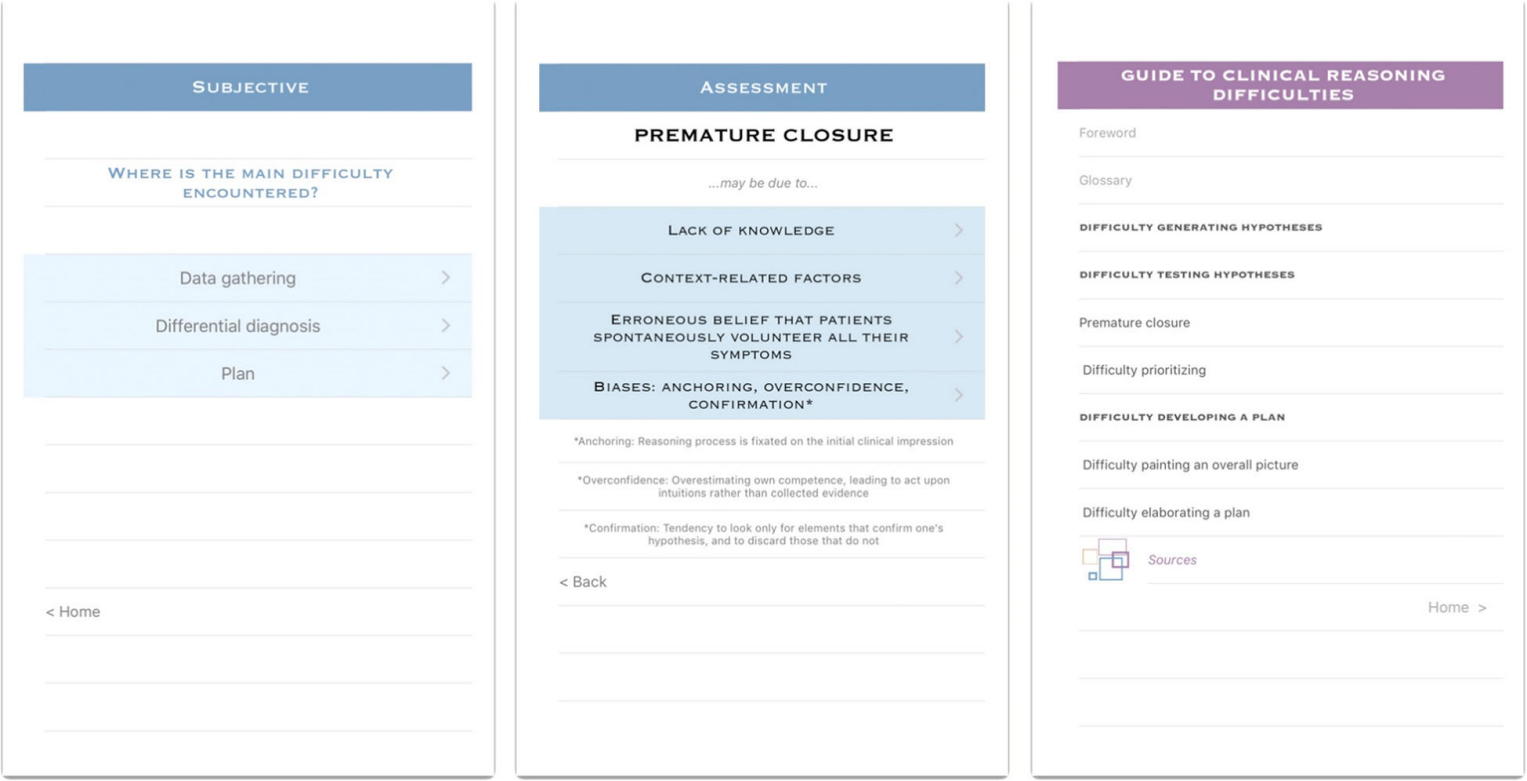
Sample screens of the Pdx application

Using XCode 7.2, we then translated our decision tree into a mobile application named Pdx (Fig. 2). To do so, we transposed junctions in the decision tree into clickable options, leading users to the next suggested step. The resulting application lets clinical teachers know what to observe when working with learners in difficulty, helps them make documented pedagogical diagnoses and suggests targeted remediation strategies. We designed a bilingual application, in English and French, bearing in mind that the language used should not be overly technical, while preserving the core pedagogical taxonomy of the reference guide. We chose to develop the application for iPhones and iPads as an initial platform, due to the prevalent use of these devices among physicians [26].

Once the beta version of the application was ready, we met the co-authors of the reference guide [8] for a roundtable, to verify the fidelity and accuracy of both the decision tree and the application. Minor changes were brought to both following this consultation, mainly with the aim of clarifying certain terms.

We then proceeded to assess the application in two academic emergency departments in Canada. We chose this setting because we anticipated that the readily accessible format of the mobile application would suit the fast pace and multiple supervisors teaching context of an emergency department. Emergency medicine is also a primary care setting where many initial diagnoses are made, which can facilitate the detection of clinical reasoning difficulties by clinical teachers.

We evaluated clinical teachers’ supervising experience with the mobile application during two semi-structured interviews, before and after a three-month trial period (November 20, 2015, to January 20, 2016). Although participation was voluntary, continuing professional development credits were offered as an incentive. The project was approved by the institutional ethics committee and written consent was obtained from all participants.

Twelve clinical teachers volunteered to participate. We first met each one individually, to document previous pedagogical experience and training, and clinical use of mobile technology (Table 1).

**Table 1:** Characteristics of participating clinical teachers

Characteristics	Number of participants
Sex	
Male	6
Female	6
Teaching experience	
Years of experience in clinical supervision	
0–5	4
5–10	3
10–15	4
15 and over	1
Previous faculty development	
None	2
1 workshop	3
≥ 2 workshops	4
Formal training	3
use of mobile technology	
Frequency of clinical use per hour	
< 1	2
1–2	6
≥ 3	4

We then briefly explained the main categories of clinical reasoning difficulties, installed the application on the clinical teacher’s device, and went through a case example. This initial interview was designed to provide a portrait of the clinical supervision context in which participants' descriptions would later be. We conducted a second set of individual interviews at the end of the trial period, to explore clinical teachers’ experience with the application. In accordance with the objectives of the study, perceived acceptability and feasibility were assessed during this second interview. The interview guide that we used for both interviews was developed specifically for this study (Additional file 1). As the interviews were conducted in French, excerpts have been translated for the purpose of this article.

This initial interview was designed to provide a portrait of the clinical supervision context in which participants’ descriptions would later be interpreted.

We analyzed the results using an interpretive description approach. A thematic coding framework was first developed by all members of the research team (EB, MCA, CSTO), based on the themes explored during the initial interviews and the adopted Van der Vleuten framework. We later expanded this first coding framework with categories emerging through open analysis of the verbatim transcripts of the interviews. We then applied the final coding framework to all transcripts (EB), using NVivo 11. We reached our final analysis and interpretations by consensus.

## Results

Clinical teachers’ overall opinion of the tool was that it was concise, concrete and easy to use. Outcomes did not correlate with previous level of experience, pedagogical training or use of mobile technology.

### Clarifying learners’ difficulties

Participants unanimously stated that the application had helped them clarify the specific difficulties of their learners. As a first step, they reported that the application had helped them validate – or invalidate – their subjective impressions that a learner was experiencing difficulties.

During the initial interviews, clinical teachers had identified their greatest challenge when supervising struggling learners as having to identify where learners’ difficulties lay.

“*The most difficult part for me is to identify where the student’s problem lies. Spotting that a student has more difficulty is pretty easy. But being able to pinpoint exactly what the problem is, I find that particularly difficult*.” P08A

Yet, when the study was over, this was precisely the aspect of the application that participants had found most helpful and they now reported feeling more confident with this task.

“*Sometimes you just get a feeling that the shift did not go very well. But this app helped me address these issues differently and ask: ‘Ok, where did it go wrong?*’” P02B

Furthermore, most participants found that the tool had helped them find the ‘right words’ to name the problem.

“*The app helped me to better define the problem, with educational words, not just: ‘You’re not good’!... It helped me find the right words*.” P01B

Being able to pinpoint difficulties helped participants give more specific, useful feedback, and helped some feel more efficient.

“*Usually, I’ll give an example and they’ll say: ‘That doesn’t count, because….’ They often find excuses and our message doesn’t seem to get through. This time when the resident said: ‘Yes, but this was because of this’, I said ‘Wait, wait, I have 3 more examples!’ I felt more much confident saying: ‘These are specifically the areas you need to work on’. The resident ended up agreeing*.” P01B

Feedback was now perceived to be more credible because clinical teachers perceived it to be reliable, and some participants reported an increased sense of competence when using it:

« *I know what I know from personal experience, but not because I was taught. So the app allowed me to offer the student something more concrete and evidence-based*. » P06B

With respect to the remediation portion of the tool, however, the pedagogical terminology appeared to be a double-edged sword. Indeed, a few participants cited the need for further examples to understand how to apply them. That being said, most participants also stated that they did not consider remediation to be part of their task during clinical supervision.

“*I never got to that part in the app. I couldn’t see myself initiating remediation strategies... We’ll give a few tips here and there, but that’s not the main part of what we do*.” P01B

### A format that facilitates learning and translation into practice

The application was considered easy to use during clinical supervision by all participants. During the three-month trial period, all but one used it in the field to solve concrete supervision issues during emergency shifts. During this period, all but two participants worked with a range of one to three learners in difficulty, which is coherent with rates of 10 to 15% of struggling medical learners reported in the literature [27, 28]. The remaining ten participants used the application on each occasion where they supervised a learner who they felt experienced more difficulty. It was estimated that using the application increased supervision time by approximately 5 min per shift, which was deemed very feasible. During the initial interviews, all participants reported consulting applications regularly to help them through various steps of their clinical tasks. It resonated therefore with their well-integrated habit of consulting mobile applications to solve clinical problems during their shifts.

Moreover, the format itself was reported to facilitate learning. Some participants mentioned that they had previously followed formal workshops on the same topics to no avail, having largely forgotten the content once the time had come to use it.

One participant had initially mentioned:

“*I attended the same workshop on clinical reasoning 2 or 3 times. It seems like each time, it remains a bit vague. It doesn’t change the way I teach*.” P08A

Yet this same participant described a different experience with the application.

*“This time, when I worked with the same student again, I was better prepared to reevaluate him. Even afterwards, with another student who had similar problems, I was better equipped to help him*.” P08B

The mobile format also allowed repetitive access to the same information, which reportedly induced effortless learning over time for some users.

“*This is the way I usually work, I learn new material by using it*.” P05B

Thus many participants felt that their need to follow the application step-by-step would decrease, as they would gradually integrate its content.

## Discussion

### Acceptability of the application

Participants responses reflected a good level of acceptability for this application, as it was mostly viewed as a helpful resource to pinpoint problems. A few participants however reported not consulting the remediation strategies section of the tool because they did not feel remediation to be part of their supervising task. Yet the clinical context is where remediation strategies are considered to be most effective [29]. This portion of the application may have elicited less acceptability because it did not reflect these clinical teachers’ perception of their role. This finding is coherent with the results of a study conducted by Audétat et al. [30]. The authors had found, consistently with the Theory of Reasoned Action, that clinical teachers were reluctant to engage in remediation strategies as a result of low self-efficacy beliefs toward remediation and a belief that their role as clinical teacher rested mainly on role-modeling, in keeping with the apprenticeship model traditionally used in medical training.

Using the application during clinical supervision was considered feasible by all participants and all but one did in fact use it during their emergency shifts. These findings are particularly relevant when interpreted in the context of emergency medicine, where time for teaching is notoriously scarce [31, 32].

### The right words to define learners’ difficulties

Participants in this study unanimously felt that the application had helped them clarify learners’ difficulties, by helping them find the right words. At the beginning of the study, being able to better document and name learners’ difficulties had been identified as an important need of the clinical teachers interviewed. This echoes Dudek et al. [6]’s finding that lack of knowledge of what to document constitutes a major barrier for reporting poor performances. Interestingly, during the post-trial interviews, participants reported that documenting and naming difficulties was what the tool had been most successful in helping them with.

Moreover, discovering the right terminology to describe learners’ performances more accurately seems to have helped clinical teachers validate whether a learner was in fact experiencing difficulties. One hypothesis to explain why this was particularly meaningful relates to the context in which feedback is given in the emergency room. Bearman et al. [1] have identified seeking a second opinion as one strategy used by clinical teachers to validate their subjective impressions. Because clinical teachers in the emergency room work one-on-one with learners who change daily, they rarely have the opportunity to discuss their impressions with colleagues who have worked with the same learner before giving feedback. In this context, the mobile tool may have been useful to provide a “second opinion” to validate clinical teachers’ initial impressions.

Although no elaborate training had been given to participants on how to use the application, all participants stated that they had found it useful to better define their learners’ difficulties. That this finding remained consistent regardless of years of experience and previous educational training suggests that the application could be used favorably for faculty development at all levels of learning. Furthermore, the application required minimal to no training for faculty to integrate it into their supervision and its deployment among faculty requires no resources. While these advantages hold true for the mobile tool in this study, we postulate that a mobile format could be used profitably in other areas of faculty development to induce self-regulated learning by clinical teachers.

### A just-in-time format mirrors clinical problem-solving practices

The mobile format provided just-in-time information at point-of-care, which seems to have acted favourably on knowledge translation and actual use of the application by participants. In addition, specific approaches to clinical reasoning difficulties were indeed used by participants during the trial period, whereas some had mentioned having followed formal workshops on this topic to no avail. The successful use of the mobile format can probably be attributed, at least partly, to the fact that consulting a mobile application to solve educational problems is coherent with clinical teachers’ well-integrated habits of clinical problem solving. Moreover, the algorithmic format of the application reflects the clinical decision rules commonly used by clinicians [33], allowing targeted information to be consulted on an as-needed basis, in response to the answers given by the user. A just-in-time solution is also coherent with principles of cognitive psychology whereby using new knowledge for problem solving is instrumental to efficient learning [34]. Finally, the mobile format allowed repetitive access to the same information, which reportedly induced effortless learning over time for some users.

### Limitations

An important limitation of this study is that all participating clinical teachers were recruited from the same university, through volunteer sampling. Thus, it is possible that their experience with the application reflected a local culture with regard to clinical teaching, or a prior interest in medical education, mobile technology, or both.

It seems reasonable to expect that this application could be found useful in a variety of clinical teaching contexts. Indeed, positive results in the busy setting of an emergency department suggest that using the application during supervision may be feasible in a wider range of clinical contexts. Moreover, the approach presented in the application is based on Audétat et al.’s guide, which itself has been validated with clinical teachers from various clinical domains, ranging from medicine to nutrition or physiotherapy [8]. Anecdotally, this same reference has also been used by one author (MCA) to guide clinical teachers in North American as well as European contexts.

Therefore, as a next step, a study should be conducted with a larger sample in a different clinical context in order to confirm our initial findings. If confirmed, the value placed on verifying impressions and finding the right words to pinpoint difficulties should be further explored in endeavours that aim to address the failure to fail phenomenon.

## Conclusion

A salient outcome in this study was that although no elaborate training had been given on clinical reasoning, all participants were still able to use the decision-support tool effectively to better define their learners’ difficulties. That this finding remained consistent regardless of years of experience and previous educational training suggests that the tool may be used for faculty development at all levels of learning. Furthermore, deployment of such a mobile tool among faculty requires no resources. While these outcomes hold true for our application in a limited setting, we postulate that a mobile format could be used profitably in other areas of faculty development to induce self-regulated learning by clinical teachers.

While the pedagogical terminology used in our tool was mostly viewed by participants as helpful to pinpoint problems, for some, terminology could also represent an obstacle that kept them from using the remediation strategies. Since a glossary was available within the application but not consulted, further development of the tool could focus on adding integrated examples and descriptions, in order to maintain the advantages of precise terms, while making them more accessible to clinical teachers. We posit, however, that the clarification of terms would not be sufficient for all clinical teachers to apply remediation strategies. While making pedagogical terminology more accessible to clinical teachers may increase their sense of self-efficacy, it remains likely that clinical teachers’ perception of their role as educators would have to be addressed for the majority of clinical teachers to engage in remediation strategies in the field, where these strategies are perceived to be most effective.

Finally, our outcomes support the use of a just-in-time and algorithmic format as an alternative medium for knowledge transfer in medical education. They suggest that optimal use of a mobile format in such contexts should combine concise, targeted and gathered information at point-of-care, and that, to be most efficient for educational purposes, the dissemination platform should mirror habitual clinical practices of intended users.

## Additional file

Additional file 1: Interview Guide. Interview guide used in the study, for the initial (t_1_) and follow-up (t_2_) interviews. (DOCX 19 kb)

## Abbreviations

CSTO: Christina St-Onge
EB: Elisabeth Boileau
MCA: Marie-Claude Audétat
