# Supplementary material for: Just-in-time faculty development: a mobile application helps clinical teachers verify and describe clinical reasoning difficulties
Source: BMC Med Educ. 2019 Apr 30;19:120. doi: 10.1186/s12909-019-1558-2 (PMC6492340; doi:10.1186/s12909-019-1558-2)
Supplement: Supplementary file 1 — Interview Guide. Interview guide used in the study, for the initial (t1) and follow-up (t2) interviews. (DOCX 19 kb) [file 12909_2019_1558_MOESM1_ESM.docx]

**INTERVIEW GUIDE**

# I- INITIAL INTERVIEW (t_1_)

**I- MOBILE TECHNOLOGY**

1. Which mobile devices do you use?
   - *iPhone*
   - *iPad or iPad Mini*
   - *iPod*
   - *Other*
2. Which of these devices do you generally carry with you during an emergency shift?
3. How often do you use these devices during a typical emergency shift?
4. For what purpose(s) may you use these devices during emergency shifts?
   - *Medical references (e.g. Pepid, Uptodate,MedCalc)*
   - *Calendar or timer*
   - *Calculator*
   - *Text messages or emails*
   - *Other*

**II- EDUCATIONAL EXPERIENCE**

1. For how long have you been supervising medical learners?
2. Do you teach medical learners in other contexts than the emergency department?
   - If so, in what contexts?
3. Have you attended or provided faculty development workshops or seminars? Please specify topic and duration.
4. Which aspects of clinical supervision do you generally find more challenging?

**III- CURRENT APPROACH TO CLINICAL REASONING DIFFICULTIES**

1. When you sense that a learner is experiencing more difficulty, do you adapt your supervision? If so, how?
2. Which possible reasons do you consider when you observe that a learner experiences more difficulty?

# II- FOLLOW-UP INTERVIEW (t_2_)

## What is your overall impression of the applciation?

1. **In what contexts did you use the application?**
   1. Only during clinical supervision? During a work shift or afterwards?
   2. To address what situations, or when confronted with which problems?
2. **ACCEPTABILITY OF THE TOOL**
3. **Did you find the application useful…**
   1. To know what to observe and what to document?
   2. To identify the underlying issue? To clarify the reasons for learners’ difficulties?
   3. To learn how to supervise learners with difficulties? To learn new supervising strategies?
   4. Which aspects of the application did you find most useful? Least useful?
4. **Did you find that the application was easy to use?**

- Why?

*Perceived feasibility of using the tool*

1. **When you used the application, did you find that it lengthened your supervision time?**

- If so, did this additional time ever keep you from using the application?

1. **Would you have any suggestions to improve the application?**
2. **Now that the trial period is over, do you think that you will keep using the application?**

- Why?
